# MSpine: a prospective longitudinal study of spinal cord lesions in multiple sclerosis: MRI monitoring and prognostic factors for active disease. A study protocol

**DOI:** 10.1186/s42466-026-00465-9

**Published:** 2026-04-17

**Authors:** Demmie Bouweriks, Daniel Kreiter, Stephanie A. M. Knippenberg, Jan G. M. C. Damoiseaux, Joost Smolders, Jeroen J. J. van Eijk, Elwin H. H. Mommers, Janet W. K. de Beukelaar, Tom B. G. Olde Dubbelink, Audrey H. H. Merry, Raymond M. M. Hupperts, Oliver H. H. Gerlach

**Affiliations:** 1 Academic MS Center Zuyd, Department of Neurology, Zuyderland Medical Center, Sittard-Geleen, The Netherlands; 2 School for Mental Health and Neuroscience, Department of Neurology, Maastricht University Medical Center, Maastricht, The Netherlands; 3 Central Diagnostic Laboratory, Maastricht University Medical Center, Maastricht, The Netherlands; 4 ErasMS, Department of Neurology and Immunology, Erasmus Medical Centre, University Medical Centre Rotterdam, Rotterdam, The Netherlands; 5 Department of Neurology, Jeroen Bosch Hospital, ’s-Hertogenbosch, The Netherlands; 6 Department of Radiology, Zuyderland Medical Center, Sittard-Geleen, The Netherlands; 7 Department of Neurology, Albert Schweitzer Hospital, Dordrecht, The Netherlands; 8 Department of Neurology, Rijnstate, Arnhem, The Netherlands; 9 Zuyderland Academy, Zuyderland Medical Center, Sittard-Geleen, The Netherlands

**Keywords:** Multiple sclerosis, Magnetic resonance imaging, Prognostic factors, Spinal cord lesions, Active disease progression, Relapsing remitting, Soluble immune checkpoint marker

## Abstract

**Background:**

Multiple sclerosis (MS) is the most common demyelinating disease of the central nervous system. Over the past years, spinal cord MRI (SC-MRI) has improved significantly in quality and has become an important part of the diagnostic workup for MS. Presently, follow-up imaging of the spinal cord is mainly performed when spinal cord related symptoms occur. However, there is increasing evidence that asymptomatic cord lesions can occur independently of brain disease activity. Even asymptomatic spinal cord lesions have been shown to be a poor prognostic factor regarding future disability accrual. Additionally, the association between (asymptomatic) spinal cord lesions and progression independent of relapse activity (PIRA) remains undefined. Prospective data on the frequency and importance of asymptomatic spinal cord lesions, and therefore the potential role of SC-MRI in treatment monitoring, is lacking.

**Objective:**

We will routinely scan the whole spinal cord (as well as routine brain MRI) to prospectively collect SC-MRI data, in addition to clinical parameters, blood-biomarkers and CSF, in recently diagnosed MS patients. The goal is to assess the incidence of asymptomatic spinal cord lesions in patients commencing disease modifying treatment (DMT) for the first time. It will be assessed how often disease activity is solely proven by SC-MRI. A secondary objective is to identify patient groups predisposed to developing new spinal cord lesions during follow-up in early disease.

**Methods:**

This will be a multicentre, prospective longitudinal, observational study. The study population consists of relapsing-remitting MS patients, between 18 and 65 years old, initiating their first DMT within 5 years of the first clinical event. We aim to include 155 patients, who will receive three yearly SC-MRI (both sagittal and axial), brain MRI and biomarker assays during follow-up of 27 months.

**Results:**

This study started in August 2024. Currently, inclusion is still ongoing.

**Conclusions:**

The goal of this study is to explore the incidence of asymptomatic spinal cord lesions in early MS and to identify prognostic factors for spinal cord disease activity.

**Trial registration:**

This study has been registered in the Dutch CCMO/OMON Registry under **NL-005846** at 12/03/2024 (https://onderzoekmetmensen.nl/nl/trial/56629) and in ClinicalTrials.gov under registration number **NCT06827834.**

## Introduction

### Background and rationale

Multiple sclerosis (MS) is an immune-mediated inflammatory disease affecting the brain and spinal cord, causing neurological complaints and physical disability. Spinal cord MS lesions, including asymptomatic lesions, have been identified as a prognostic factor for conversion of a clinically isolated attack to clinically definite MS, for development of progressive MS and for higher levels of sustained clinical disability in the long term [2, 22]. It is debatable whether generalised and widely used study findings on prognostic markers and treatment effect using brain MRI outcomes in MS can be extrapolated to the spinal cord. Several observations support this doubt:

Firstly, the spinal cord differs from the brain, not just anatomically (volume, white/grey matter organisation, vascularization), but also in barrier functions; the composition and function of the blood-spinal cord barrier are distinct from the blood-brain barrier. Secondly, immunological characteristics (certain lymphocyte subsets and cytokine profiles which influence localization of inflammatory activity) may also differ in the spinal cord. Last- and most importantly, MRI studies suggest that brain and cord pathology radiologically progress independently [13].

In clinical practice, spinal cord MRI (SC-MRI) is mostly performed in case of possible cord-related symptoms or unexplained clinical progression, while brain MRI is performed in standard follow-up to assess radiological progression of disease. Although most spinal cord lesions are clinically symptomatic, it was reported that new spinal cord lesions may occur in absence of clinical signs or symptoms (31.2% of all reported SC-lesions), even independently of brain MRI activity (12.1%) [8]. Additionally, little is known about the effect of disease-modifying treatment (DMT) on spinal cord lesions [13].

A commonly used outcome measure in MS drug trials is ’no evidence of disease activity’ (NEDA). NEDA-3 is defined as no relapses, no disability progression, and no new radiological activity on MRI. Regarding the latter, in almost every MS drug trial, only brain and not SC-MRI is included [16], with retrospective studies showing contrasting results regarding the value of adding SC-MRI in NEDA evaluation [6, 21]. However, inherent to the retrospective design of these studies, they suffer from selection bias. In a study investigating NEDA in a large cohort of MS patients using first- as well as second-line treatments, every year there were 8% − 12% more patients showing evidence of disease activity solely based on SC-MRI [20].

A recently introduced concept in MS is progression independent of relapse activity (PIRA) and/or progression independent of relapse and MRI activity (PIRMA), highlighting the significance of clinical disability progression that occurs regardless of relapse events or MRI findings. PIRA/PIRMA represents the proposed pathological mechanism of chronic neurodegeneration in MS as opposed to acute inflammation associated with relapse.

Evidence on the contribution of spinal cord lesions, particularly asymptomatic lesions, to PIRA/PIRMA remains scarce, largely due to the omission of routine spinal cord imaging in standard MRI protocols. Given that asymptomatic spinal cord lesions have been implicated in disability progression, insufficient monitoring of the spinal cord may lead to an underestimation of their potential role in the PIRA/PIRMA mechanism [4]. The operational definition of PIRMA may possibly require expansion to incorporate routine spinal cord MRI monitoring, thereby capturing asymptomatic spinal cord lesions that could otherwise confound assessments of progression independent of relapse and (brain) MRI activity.

**Fig. 1:**
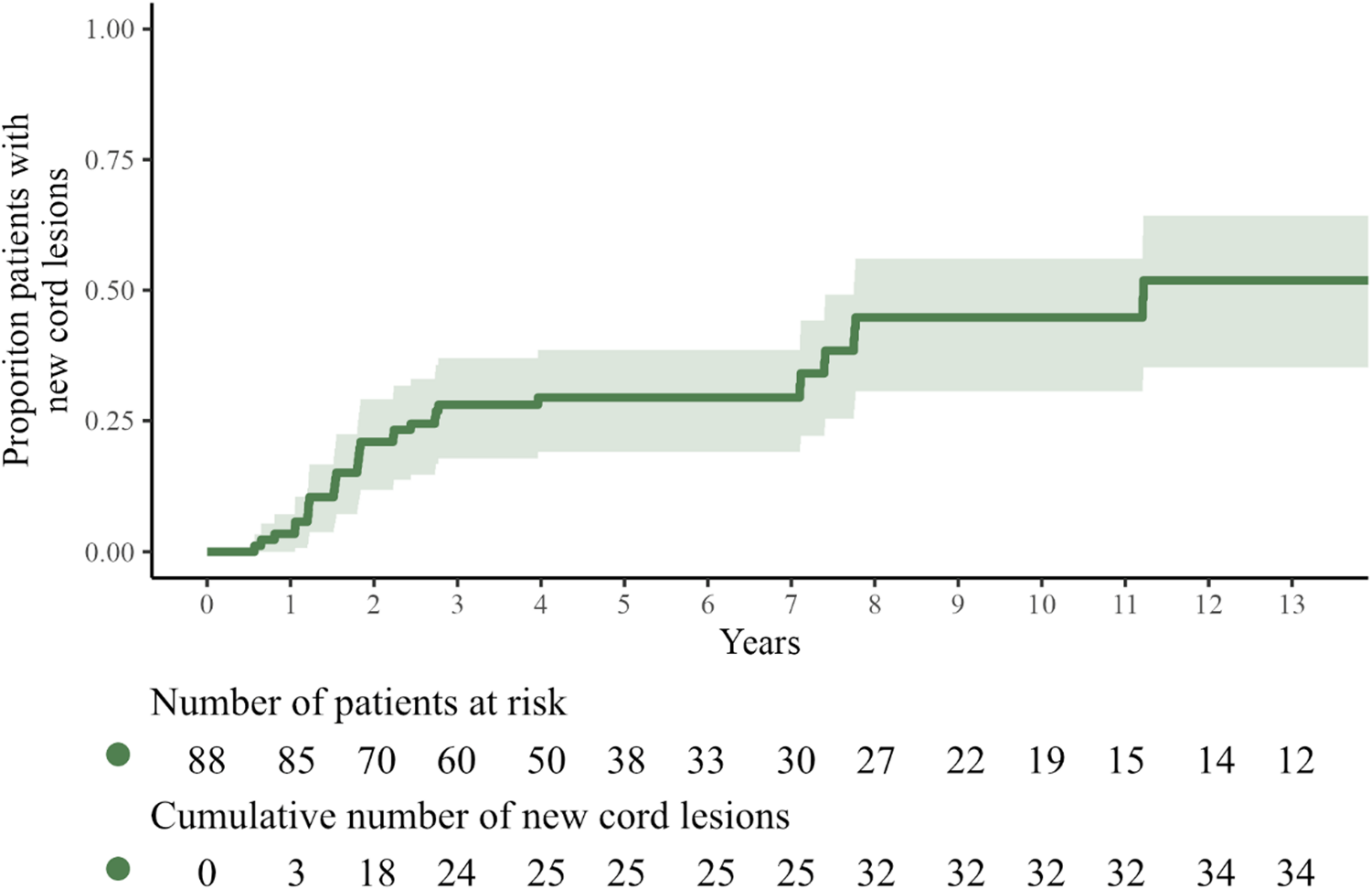
New spinal cord lesions in patients receiving DMT (> 90% of follow-up duration low-efficacy DMT or > 90% of duration high-efficacy DMT) from a retrospective cohort (Zuyderland MC) of patients that underwent > 1 SC-MRI during follow-up. Adapted from Kreiter et al. [11].

We reported previously in a retrospective setting, that approximately a quarter of patients under a DMT developed new spinal cord lesions in the first 3 years after starting treatment (see Fig. 1) [11]. Other reports estimate that about 10–25% of spinal cord lesions are asymptomatic and therefore are missed when SC-MRI is not part of routine follow-up after starting a DMT [3, 22].

Given that (asymptomatic) spinal cord lesions may occur with some frequency, possibly remain undetected in current follow-up regimens, and are not accounted for in the outcome measures of clinical drug trials—yet may have implications for future disability—an important question arises: should routine spinal cord follow-up be incorporated into standard clinical practice?

This study seeks to determine the frequency of asymptomatic spinal cord lesions in patients with early MS initiating their first disease-modifying treatment, and to evaluate whether spinal cord MRI detects disease activity not captured by clinical assessment or brain MRI alone.

### Spinal cord-disease prone subgroups

As described above, adding SC-MRI to routine follow-up in MS patients could bring several advantages and opportunities. However, incorporating routine spinal cord imaging into follow-up protocols for all MS patients presents several disadvantages. It significantly prolongs the scanning protocol, with consequently adding considerable costs and burden on the often-scarce MRI scanning capacity. Furthermore, extended MRI scanning times impose a greater burden on patients. Ideally, subgroups can be identified that are prone to more spinal cord involvement and that would benefit from routine spinal imaging. To this end, increasing our understanding of factors that contribute to spinal cord pathology in MS is needed.

Currently, the relationship between baseline patient characteristics and radiological, biochemical, and genetic markers with new spinal cord disease activity remains poorly defined. Cross-sectional studies have demonstrated some associations: for instance, certain cerebrospinal fluid (CSF) profiles—particularly intrathecal IgM synthesis—have been linked to spinal cord lesions. Whether intrathecal IgM synthesis also predicts more spinal cord involvement at follow-up is unknown. Additionally, MS patients with the HLA-DRB1*1501 allele show more extensive spinal cord pathology in cross-sectional studies [13].

From a clinical perspective, the initial presenting syndrome, such as optic neuritis or myelitis, may be relevant for identifying patients with a higher likelihood of significant future spinal cord involvement. From a radiological standpoint, the presence of spinal cord lesions early in the disease course could serve as a potential indicator of spinal cord-predominant disease and may warrant consideration for routine spinal cord imaging follow-up. Other markers that could be of interest in the context of selecting patients with risk of spinal cord demyelination are blood markers that have previously been associated with an increased risk of disease progression. The specific relation of these markers to spinal cord demyelination is yet to be determined.

The following blood biomarkers are specifically of interest; low baseline 25-hydroxyvitamin D level [1], high neurofilament light chain (NfL) levels and glial fibrillary acidic protein (GFAP) [5], which have been associated with disease activity on cerebral MRI and disability progression.

Additionally, plasma-based immunological biomarkers could be promising candidates. For example, within the group of soluble immune checkpoint markers (sICPs) multiple markers, such as soluble IL-2, soluble CD27, soluble CD14, soluble B cell maturation antigen, soluble TREM-2, have been identified as elevated in MS patients and correlate with signs of clinical and/or brain MRI disease activity [14, 15]. Their association with spinal cord lesions is not yet known.

It was also found that patients treated with interferon-beta without brain MRI activity tend to have a favourable level of transitional B cells [17], but their association with progression/activity on SC-MRI is to be determined. From experimental autoimmune encephalomyelitis (EAE) experiments there is evidence that the balance between CD4 + T cell subsets and their cytokine profiles influence spatial localization of inflammatory activity. The balance between Th1 and Th17 cells is thought to determine whether there is more brain or spinal cord inflammation, foremost mediated by IFN-γ and IL-17 [7], suggesting that immunological markers are possible candidates for predicting more spinal cord involvement.

## Methods

### Aim of the trial

The primary objective of this study is to prospectively collect both full sagittal and axial SC-MRI data (in addition to routine brain MRI), in recently diagnosed MS patients, to address the following research questions:

What is the incidence of asymptomatic spinal cord lesions in patients commencing DMT? How frequently do asymptomatic spinal cord lesions occur in absence of radiological progression on brain imaging? In other words, how often is disease activity solely proven by SC-MRI and what is the number-needed-to-scan?

The secondary objective is to investigate which patients are predisposed to developing new spinal cord lesions during follow-up in early disease stages. Regarding this question, the primary focus will be on factors such as CSF profiles, lymphocyte composition in blood, blood biomarkers and clinical features.

We hypothesize that the detected incidence of asymptomatic cord lesions independent of brain MRI activity will be higher than the approximately 10% per year reported in retrospective studies [8, 20]. Additionally, we expect that more SC lesions occur in a subgroup of MS patients with CSF profile positive for intrathecal IgM, IgG synthesis and higher number of oligoclonal bands, a low number of transitional B cells at baseline, increased or decreased levels of sICPs at baseline, and/or a presenting clinical syndrome of optic neuritis or myelitis.

### Study description and study design

#### Outcomes and participant timeline

The follow-up period will be 27 months and visits will take place at approximately 3, 15 and 27 months. At these follow-up moments, next to routine yearly brain MRI, spinal cord coverage will be added to the scan protocol.

**Fig. 2:**
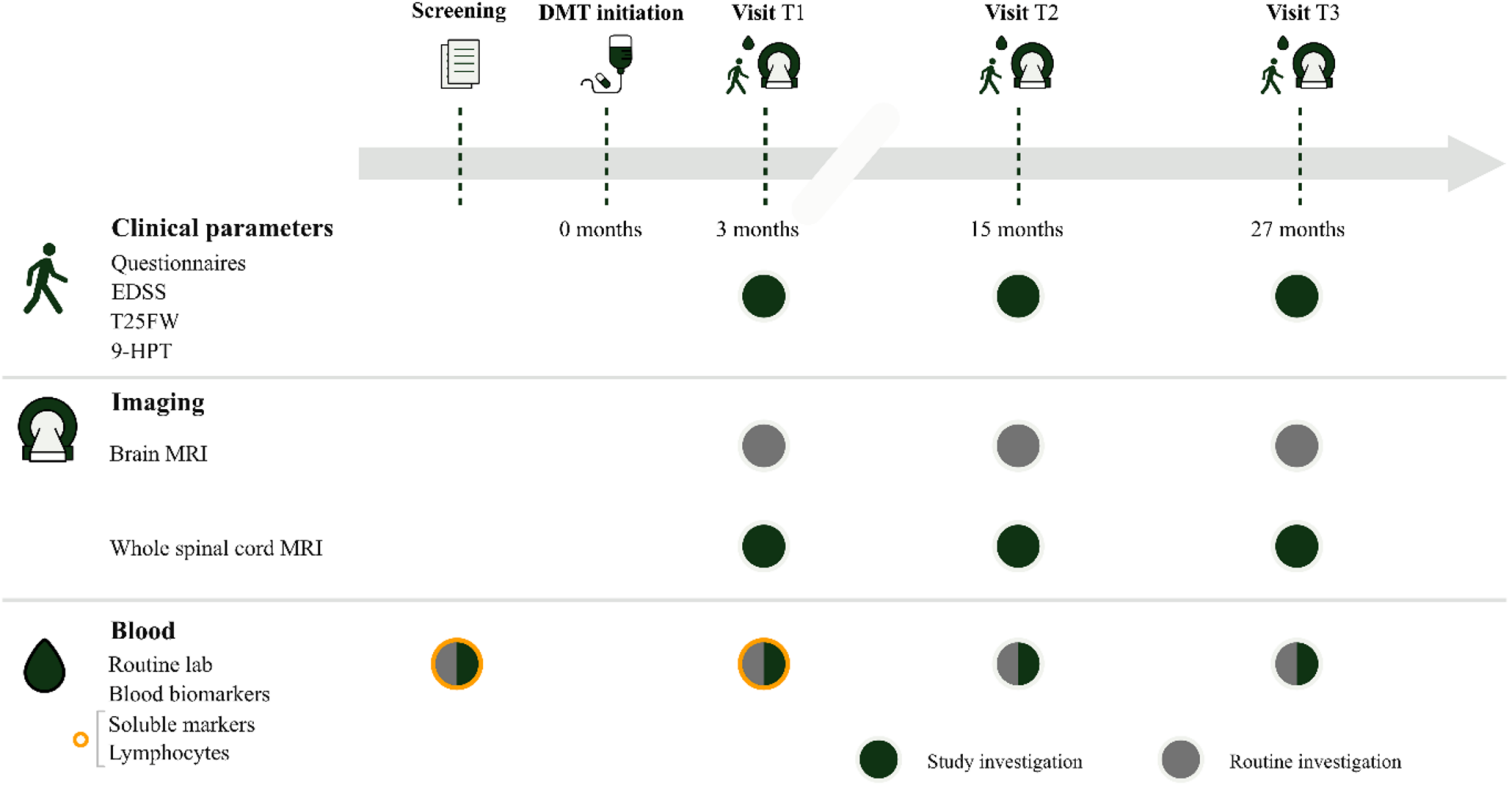
Overview of study timeline and collected data. See supplementary material for additional concise information

During the follow up visits, routine consultation with a standard medical history, a questionnaire regarding potential urinary tract symptoms, additional clinical tests/parameters, and additional blood collection will be performed. Figure 2 gives a concise overview of study procedures during the follow up period.

*At baseline*,* the following characteristics will be registered: age*,* sex*,* height and weight (body mass index)*,* comorbidities*,* cerebrospinal fluid data (if lumbar puncture took place as part of the diagnostic workup)*,* presenting syndrome*,* disease duration*,* family history of MS*,* previously performed cerebral and SC-MRI data.*

*Furthermore*,* MS related relapses*,* extra outpatient clinic visits*,* clinical testing and performed MRI scans of brain or spinal cord will be registered in the study as additional visits and scans*.

### Outcome measures

Full inclusion is sought to be achieved by mid-2026 which will result in the ending of the data collection mid-2028. It is expected that data analysis and subsequent publication of results will follow in the second half of 2028. The following study parameters are measured during study conduction.

#### MRI

During follow-up the SC-MRI-scans are performed in addition to the brain MRI. As explained in Table 1, MRI protocol will contain sagittal as well as axial sequences of the complete spinal cord. MRI-images will be assessed and reported by a local radiologist of the participating centres for clinical purposes. The images and reports will then be exported and pseudonymised and are then reviewed by a second neuroradiologist who is part of the study team. The primary and secondary reports will be compared to assess interobserver reliability. If first and second reports differ, a third neuroradiologist will be consulted for arbitration after assessment of the previous reports and images. Gadolinium-based contrast agents will not be used for routine follow-up imaging, in accordance with current guidelines. Contrast enhancement rarely adds clinically relevant information for longitudinal assessment, does not change therapeutic decision-making, may even influence volumetric measurements, and carries cumulative exposure considerations (Pawlitzki et al., [18, 19].

**Table 1:** MRI protocol

MRI protocol	Brain MRI	Spinal Cord MRI
Scanner	Preferably 3 T MRI (if unavailable, the same MRI scanner, e.g. 1.5T, should be consistently used per subject)	
Sequences	Local routine MS follow-up brain protocol including at least an axial FLAIR	**Sagittal**: T1 (3 mm), T2 (3 mm), STIR (3 mm) (Cervical, Thoracic, Conus Medullaris) **Axial**: T2* MEDIC (3 mm) [C0-C7], T2 TSE (4 mm) [C0-L1]

In line with current MS diagnostic and treatment guidelines, all patients will have had a recent brain and SC-MRI prior to DMT initiation, either diagnostically or due to disease activity prompting treatment. These pre-treatment MRIs will be collected retrospectively and included in the analysis.

#### Blood tests

In routine clinical practice, blood samples are collected from patients prior to treatment initiation to assess parameters such as white blood cell count, liver and renal function and vitamin D levels. These assessments are repeated during regular follow up. In addition, serum levels of NfL and GFAP will be measured at screening, T1, T2 and T3. Circulating sICPs and lymphocyte subset distributions will be analysed in screening and T1.

#### Clinical tests

In addition to routine patient visits, at screening, T1, T2 and T3 the following clinical tests are conducted:

- Extended Disability Status Scale (EDSS): EDSS is used to determine disability worsening and assess relapse related decline in neurological function.
- Timed 25-foot walking test (T25-FW): The T25-FW is a quantitative measure to assess mobility and lower leg limb function.
- 9 Hole Peg Test (9HPT): The 9HPT is a manual dexterity performance measure and assesses disability in the form of upper limb function.

A questionnaire is administered at screening, T1, T2, T3 as well and considers urinary tract symptoms. They consist of a separate questionnaire for male (ICIQ-MLUTS) and female (ICIQ-FLUTS).

### Recruitment and eligibility criteria

We aim to include a population of treatment-naïve RRMS patients, 18–65 years old, early in the disease course, when a first DMT is initiated. Inclusion is performed in five Dutch MS centres; Zuyderland Medical Centre, Erasmus Medical Centre, Jeroen Bosch Hospital, Rijnstate Hospital and Albert Schweitzer hospital. In- and exclusion criteria have been explained in detail in Table 2.

**Table 2:** In- and exclusion criteria

Inclusion Criteria	Exclusion Criteria
Diagnosed with RRMS (≤ 5 years since first clinical event)	First clinical event > 5 years ago
Age between 18–65 years	Age < 18 or > 65 years
Treatment-naïve patients starting a currently approved DMT in the Netherlands	Prior DMT initiation
	Incapable of giving informed consent
	Unable to undergo local MRI scan - Physical limitations (e.g., obesity, inability to lie flat) - Claustrophobia
	Contraindications for MRI scan - MRI-unsafe implants/devices (e.g., pacemaker, ocularmetal splinters) - Pregnancy at inclusion

Individual subjects will not be replaced after withdrawal. Sample size calculation has been done with correction for expected loss to follow up. This study does not include any interventions. Patients who choose to withdraw from the study will go back to receiving standard care.

### Statistics

#### Sample size

To estimate the incidence of asymptomatic cord lesions in absence of brain MRI activity, an approximate sample size of 139 will be needed, given an estimated proportion of asymptomatic cord lesions of 10% based on retrospective literature [6, 8, 20] (precision 0.05, confidence level 0.95).

CSF profiles are studied as a priori selected predictor and additionally we provide descriptive data and analyse associations in an exploratory fashion for the other different clinical, radiological, biochemical factors and treatments used in patients with/without new spinal cord lesions, to be able to identify important prognostic candidates, which in turn can be specifically studied in a possible future extension of the study.

Not all collected parameters are included in a prognostic model for spinal cord disease activity, because for the prognostic model to include a wide variety of predictors, a very large population is needed (estimated > 400), which currently is not feasible.

To assess a logistic regression model in which certain CSF profiles (primarily the degree of intrathecal IgM production) are associated with any new spinal cord lesion within two years of follow-up, an estimated sample size of 144 patients is needed to be able to detect an odds ratio of 2 (expected R-squared between main predictor variable and other covariates estimated 0.07 based on retrospective data) with a power of 80%, alpha of 0.05 given that the base expected proportion of patients with new cord lesions within two years is about 15%. The latter figure is based on retrospective data from a local database, where we found that 19% of patients (*n* = 217) developed new cord lesions (regardless of treatment) within 2 years of initiation of a new treatment [11]. In the international MSBase registry this figure was 12% for treatment-naïve RRMS patients starting a DMT (*n* = 528) [12].

Given the calculated sample sizes and an expected loss-to-follow-up of about 2–7% based on other longitudinal prospective MS MRI studies [9, 10], for this study we will aim to include 155 patients.

### Statistical analysis

#### Primary analysis

The primary objective is to determine the incidence of new asymptomatic spinal cord lesions in the study population. The incidence of new asymptomatic spinal cord lesions will be calculated as the proportion of patients with at least one new lesion at the predefined follow-up timepoints. 95% confidence intervals (CI) will be calculated using exact binomial methods. The relationship between new asymptomatic spinal cord lesions and concurrent brain MRI activity will be assessed to identify lesions occurring independently of brain activity. From these data, the *number needed to scan* (NNS) will be calculated as the inverse of the proportion of patients with isolated asymptomatic spinal cord activity detected at 15 months (T2) and 27 months (T3) after DMT initiation.

#### Secondary analysis

The association between CSF profiles and the occurrence of any new spinal cord lesion within two years (binary outcome) will be evaluated using a multivariable logistic regression model with intrathecal IgM synthesis (standardized to z-scores using Reiber formula) as the primary predictor. Prespecified covariates are intrathecal IgG synthesis, presence of oligoclonal bands and kappa free light chains. Continuous predictors will be assessed for linearity in the logit and transformed or modelled with splines if needed, multicollinearity will be evaluated using variance inflation factors. Results will be reported as odds ratios with 95% CI. Model discrimination will be quantified by the area under the ROC curve. Distributions of lymphocyte subsets, soluble blood- and blood biomarkers, and clinical variables will be compared between groups with and without new SC-MRI activity, overall MRI activity, and NEDA status using independent *t*-tests or Mann–Whitney *U* tests for continuous variables and χ² tests for categorical variables. Exploratory analyses will compare other baseline characteristics (e.g., center of inclusion, disease duration, treatment choice) across lesion-based subgroups.

## Perspectives

Despite significant advancements in the management of MS in recent years, the disease remains one of the leading causes of non-traumatic disability in (young-) adults worldwide. There is a pressing need for prognostic markers to better predict disease progression and guide therapeutic interventions. Spinal cord lesions play a critical role in the trajectory of MS and contribute substantially to long-term disability accumulation. However, there are still a lot of uncertainties in regard to follow-up imaging of the spinal cord, which is the core subject of our research. Spinal cord imaging is costly and time-consuming; therefore, it is not efficient to simply add this to the existing brain imaging follow-up protocol. To that end, we are assessing prognostic factors for future spinal cord lesions such as clinical, cerebrospinal fluid and blood biomarkers to identify subgroups of patients that would benefit from regular spinal cord imaging in follow up.

Currently, our knowledge is limited regarding (i) the determinants of spinal cord involvement, (ii) which patients would benefit from more extensive spinal cord MRI follow-up and (iii) how we should treat patients with a greater degree of cord disease activity (the latter knowledge gap falls outside the scope of the present study but could give first insights and provide basis for further research).

Retrospective research provides evidence for the importance of asymptomatic spinal cord lesions on the disease course in MS patients, as well as possible spinal cord disease-prone subgroups. However, prospective research is lacking. The design of this study aspires a better understanding of asymptomatic spinal cord involvement in MS patients and insight into spinal cord-disease prone subgroups of MS patients.

With this study, the goal is to:

1. Improve understanding of the incidence of asymptomatic spinal cord lesions, particularly how often they occur without accompanying progression on brain MRI.
2. Improve spinal cord imaging monitoring strategies by gathering data on the relevance of these lesions. This includes identifying markers of subgroups prone to spinal cord involvement, which may warrant more tailored monitoring protocols.
3. Support the optimization of personalised monitoring approaches and in turn enhanced personalised treatment approaches, with the ultimate aim of reducing disease progression. Improved monitoring can enhance clinical decision-making and potentially have a meaningful impact on patients’ lives, including their level of disability, psychological well-being, and social participation.

Furthermore, this topic should be the focus of future research, as it has been largely underrepresented in current prospective scientific discourse. At present, there is a significant knowledge gap within this area, and this study seeks to address part of this uncertainty by shedding light on its implications for both patient monitoring, care and future investigations.

The findings are expected to give a renewed insight into the incidence of asymptomatic spinal cord lesions, and the importance of follow-up MRI of the spinal cord in (a subpopulation of) MS patients.

## Data Availability

With the publication of the results, we aim to publish the dataset in a scientific data repository compliant with the FAIR data principles.

## Abbreviations

9HPT: 9-Hole Peg Test
ABR: General Assessment and Registration form (ABR form), the application form that is required for submission to the accredited Ethics Committee; in Dutch: Algemeen Beoordelings- & Registratieformulier (ABR-formulier)
CA: Competent Authority
CCMO: Central Committee on Research Involving Human Subjects; in Dutch: Centrale Commissie Mensgebonden Onderzoek
CIS: Clinically Isolated Syndrome
CSF: Cerebrospinal Fluid
EDSS: Expanded Disability Status Scale
IC: Informed Consent
MRI: Magnetic Resonance Imaging
sICP: soluble immune checkpoint markers
T25-FW: Timed 25 Foot Walking Test
